# Parenting, health, and use of medications among college youth: The PHARMACY survey study

**DOI:** 10.1016/j.pmedr.2021.101623

**Published:** 2021-10-25

**Authors:** Caitlin C. Abar, Alexis Combs, Allison Miley, Rebecca Ruffino, Beau Abar

**Affiliations:** a The College at Brockport, Brockport, NY, USA; b University of Rochester Medical Center, Rochester, NY, USA

**Keywords:** Prescription, Opioids, Antibiotics, Parenting, College

## Abstract

Prescription medication misuse represents a major public health concern, with high rates of negative consequences observed among youth. The current study examines the potential roles parents can play in mitigating youth prescription-related harm, with a particular focus on opioid and antibiotic safety. A sample of 167 students from a mid-sized college in the Northeast U.S. were surveyed on their prescription medication knowledge, comfort, and behaviors and perceptions of their parents’ behaviors. Results showed that parenting behaviors like targeted discussions on prescription medication safety (e.g., proper disposal of excess medication) were associated with greater medication-related comfort, which, in turn, was associated with safer prescription medication behaviors.

## Highlights

• Parent-youth conversations can improve youth comfort with prescription medications.
• Comfort with medication disposal is associated with safe and appropriate disposal.
• Specific conversations matter, in some cases, more than general parenting behaviors.

## 1 Introduction

In 2017, an estimated 18 million people in the United States have misused medications at least once in the past year (Center for Behavioral Health Statistics and Quality, 2017). Studies suggest that college students are some of the most vulnerable for misuse. Prescription painkillers, such as Vicodin, Percocet, and Oxycontin, are the second most commonly misused prescription medication among college students, behind only prescription stimulants commonly used for enhancing focus (Cutler and Kremer, 2017). One study found over 27% of young adults admitted to using prescription opioids in the previous year, and approximately 1 in 20 adolescents and young adults reported either an opioid use disorder or nonmedical use of prescription opioids in the last year (Hudgins et al., 2019). In the same study, three-quarters of those who reported nonmedical use of prescription opioids got them from outside the healthcare system (Hudgins et al., 2019). A recent *meta*-analysis focused specifically on college students demonstrated an estimate of opioid misuse between 1 in 25 and 1 in 5 (Weyandt et al., 2020). Consequences of opioid misuse are profound, as approximately 1 in 20 deaths of 12–19 year olds in the United States are due to accidental poisonings including medication overdoses (Blum and Qureshi, 2011).

In addition to opioids, antibiotic misuse poses a major threat to public health. Specifically, overuse and misuse of antibiotics has led to an antibiotic resistance crisis. Annually in the United States, 2.8 million people obtain resistant bacterial infections resulting in roughly 35,000 deaths (CDC, 2019a). One component of the antibiotic resistance crisis is antibiotic over-prescription. A population based analysis in the U.S. in 2010–2011 found that 12% of all outpatient visits resulted in an antibiotic prescription, of which approximately 30% were unnecessary (Fleming-Dutra et al., 2016). Failing to complete a course of antibiotics is also problematic, as it can result in resistant infections. An international study from 9 countries found that 1 in 4 patients saved part of their prescribed antibiotics for future use (Pechère, 2001). This practice can further exacerbate resistance risks. These risks are often underappreciated, as research estimates that 58% of patients are unaware of the potential health dangers that come with antibiotic misuse (Vanden Eng et al., 2003), and approximately 1 in 5 consumers expect an antibiotic prescription for a viral cold or flu on which the medication will have no effect. (Gaarslev et al., 2016). One study found that no participants mentioned antibiotic resistance as consequence of taking antibiotics inappropriately, and when the issue was brought up, they did not realize that they personally could impact or be affected by it (Gaarslev et al., 2016). As a result, patients frequently view taking antibiotics as a better alternative than not taking them because they believe there remains a chance they might prove helpful. (Broniatowski et al., 2015). Having this mindset is dangerous because it supports the common misconception that antibiotics can be taken without risk.

Adolescents and young adults are a significant part of the prescription drug misuse issue. In the 2012 Partnership Attitude Tracking (PAT) Study, 25% of teens reported misuse of a prescription medication in their lifetimes. What is even more concerning is the growth of these behaviors. From 2008 to 2012, prescription drug misuse by teens increased by 33% (PDFK, 2013). In regard to antibiotics, a study at a university student health center targeted students due to (a) their higher susceptibility to respiratory infections and (b) their elevated need to return to their daily activities frequently perpetuates their desire for prescription medication (Haltiwanger et al., 2001). The results of the study showed that 55% of college students expected an antibiotic prescription going into their visit, and satisfaction was more likely when the student received an antibiotic prescription (Haltiwanger et al., 2001). These results are relevant because students are often misinformed and expect an antibiotic prescription regardless of whether their illness indicates one.

Several efforts and interventions to curb prescription medication misuse have been implemented. In particular, an NIH-funded study found that middle school students from small towns who participated in a community-based prevention program were less likely to abuse prescription medications in late adolescence and early adulthood than those who did not (Valdez, 2014). Additionally, the prescription drug monitoring program is currently in 48 states, which is an electronic database that tracks prescriptions and can potentially recognize those who are at risk for misusing prescriptions (Valdez, 2014). Finally, a study found that patients who were prescribed opioids greatly benefited from 15-minute Script Safety, knowledge on safe prescription opioid use, storage and disposal improving significantly and sustained at a month follow up (McCauley et al., 2013).

Antibiotic stewardship efforts in health care have been made encourage appropriate prescribing behaviors to limit resistance (Sanchez et al., 2016). In 2013, approximately 67 million antibiotics were prescribed to patients younger than 19 (CDC, 2019b). Additionally, young adults visited the emergency department for antibiotic-related adverse drug events from oral antibiotics twice as often as adults over 65 years old (CDC, 2019b). One method that has been explored for combatting antibiotic misuse in adolescents involved educational handouts and verbal instruction from healthcare providers about antibiotics on college campuses (Moes et al., 2018). On a national level, the Centers for Disease Control and Prevention (CDC) recognizes that using antibiotics in healthcare increases the development of antibiotic resistance and considers this a global health problem (CDC, 2013). The CDC declares that identifying and countering antibiotic resistance requires implementing the One Health approach to data collection and using evidence-based infection control practices to prevent the spread of resistance (CDC, 2013).

Parents play a major role in youth prescription medication knowledge and behaviors. Qualitative interviews of parents with teen children revealed that parents often have the misconception that their children cannot get high from prescription drugs and that their kids would not be interested in them. Consequently, most parents kept prescription drugs in their homes and failed to take precautionary measures to prevent teens from accessing them (Friese et al., 2013). One study found that 56% of teens state that it is easy to access prescription medication from their parents’ medicine cabinets (PDFK, 2013). An additional study that investigated parents antibiotic use in three Chinese provinces found that almost half of participants reported keeping leftover antibiotics at home for children (Sun et al., 2019). A recent review in the U.S. similarly noted that a 2018 national internet survey of parents found 48% of parents kept leftover antibiotics for future use (Grigoryan et al., 2019). In a study of 8th and 9th graders, of those who were prescribed medications in the pain, stimulant, antianxiety, or sedative categories, almost 3 of every 4 (74%) reported having unsupervised access to medications with possibility for misuse (Ross-Durow et al., 2013). Limiting access to medications is recognized as vital toward ensuring youth safety, as is teaching youth the importance of medication safety (see the CDC’s The Prevention of Overdoses and Treatment Errors in Children Taskforce Initiative; https://www.cdc.gov/MedicationSafety/protect/protect_Initiative.html).

More general parenting characteristics have been linked with appropriate prescription medication outcomes among youth. Greater parental monitoring and warmth was associated with having less favorable attitudes toward and fewer friends supportive of nonprescription use of opioids and stimulants (Donaldson et al., 2015). Another study found that adolescents who had strong family and school bonds are less likely to report misusing prescription drugs (Ford, 2009). Overall, in order to prevent prescription drug misuse, communication between parents and children may be key. The PAT study revealed only 15% of teens reported discussing prescription drug misuse with their parents (PDFK, 2013). The lack of conversation surrounding prescription medications becomes even more alarming when comparing this statistic to the approximately 80% of teens who reported having a conversation about alcohol and marijuana with their parents (PDFK, 2013).

### 1.1 Study objective

The current study seeks to expand upon existing literature by examining parental influences, both general and domain specific, on youth prescription medication knowledge, comfort, and behaviors.

## 2 Methods

### 2.1 Participants and procedure

A total of 167 undergraduate students at < BLINDED FOR REVIEW*>,* a mid-sized college in the Northeast U.S., participated in this study. The average age of participants was 18.74 years (SD = 1.16), and the majority was female (n = 118; 71%). A large majority of participants described their sexual orientation as straight (n = 148; 89%). The average amount of semesters participants had completed at < BLINDED FOR REVIEW > was 1.3 semesters (SD = 1.71). Nine of the students had transferred to < BLINDED FOR REVIEW > from another two year/four year college (5%). With regard to racial background, 136 participants identified as White (81%), 17 identified as Black/African American (10%), and 12 participants identified as Hispanic (7%). At the time of the study, all participants were enrolled in an Introduction to Psychology course and received partial course credit in exchange for their participation in this online survey study. The most common college majors among this sample were nursing (n = 50), psychology (n = 23), and criminal justice (n = 22). The institutional review board at < BLINDED FOR REVIEW > provided approval for this study, and each participant provided informed consent.

### 2.2 Measures

#### 2.2.1 Prescription medication use

Students were asked if: (a) they are currently on any prescriptions, (a) how long they have been on a prescription (greater than6 months, 6 months-1 year, 1–2 years, or 2 + years). These variables were combined for analytic purposes to represent recent prescription medication history: 0 = none to 4 = 2 + years.

#### 2.2.2 Prescription medication behaviors

Students answered the following questions regarding antibiotics and opioids separately: (a) In the past, have you ever not finished an antibiotic/opioid prescription as intended? (b) Do you currently have any excess antibiotics/opioids in your house/apartment/dorm/etc.? (c) Have you ever used excess antibiotics/opioids for an illness that it was not prescribed for? (d) Have you ever provided anyone else with your excess antibiotics?

#### 2.2.3 Prescription medication comfort

Students self-reported, on a scale from 1 to 10 (with 1 meaning not at all and 10 being 100% comfortable), “How comfortable are you with a) following prescription guidelines? b) filling a prescription on your own? c) disposing of medications when no longer needed?”

#### 2.2.4 Parent-child communication about prescriptions

Students were asked to report on did your parents ever talk to you about (a) prescription medication safety? (b) taking your prescription medication as prescribed? (c) what to do with excess/leftover medication?

#### 2.2.5 Parent-child relationship quality

Mother-child and father-child relationship quality were indexed using 6 items from the National Longitudinal Survey of Youth 97 (α _mother_ = 0.95; α _father_ = 0.92). Students were instructed to answer these questions with respect to their primary parental figures. These scales have been used previously in studies on youth substance use (Abar et al., 2014), educational attainment (Orthner et al., 2009), and delinquency (Walters, 2019).

#### 2.2.6 Parental active tracking

Parental behavioral efforts to track the activities of their college-age children were indexed using a 9 item scale developed for this population by Abar and colleagues (2019). Items were measured on a five point scale from 1 = never (0%) to 5 = yes, always (100%) (α = 0.75). This scale has previously been found to be associated with college student dietary behaviors, exercise frequency, and sleep hours (Abar et al., 2021).

### 2.3 Plan of analysis

Standard descriptive statistics (mean, standard deviations; frequencies and percentages) were used to summarize the demographic and behavioral characteristics of the participants. Youth perceptions of comfort with prescription-related activities were compared using paired-sample t-tests. Comfort variables were then predicted by demographic and parenting characteristics using multiple linear regression. Follow-up analyses examining prediction of youth prescription-related behaviors were performed using multiple logistic regression, with associations detailed using odds ratios. A p-value<0.05 was used to indicate statistical significance, with values between 0.05 and 0.10 approaching significance.

All analyses were performed in IBM SPSS version 25.

## 3 Results

### 3.1 Descriptive statistics and preliminary analyses

Approximately two-thirds of participants were not currently taking any prescription medications (*n* = 108; 65%), though the majority who were taking medications had been on them for longer than 6 months (*n* = 47; 80%). A total of 64 participants reported not finishing an antibiotic as prescribed (38%), while 12 participants did not finish an opioid prescription as intended (7% overall; 26% of participants who had been prescribed an opioid). Excess antibiotics were present in 16% of participant residences (*n* = 26), and excess opioids were present in 10 participant residences (6% overall; 15% of participants who had been prescribed an opioid). A total of 18 participants reported taking excess antibiotics for an illness that they were not prescribed for (11%), and 5 provided excess antibiotics to someone else to use (3%). A total of 6 participants reported taking excess opioids for something other than for what it was prescribed (4% overall; 7% of participants who had been prescribed an opioid), though only 1 provided excess opioids to someone else to use (<1% overall).

In general, students reported high levels of comfort following prescription guidelines and moderate levels of comfort with filling a prescription on their own and disposing of medications when no longer needed (see Table 1). Paired-samples t-tests demonstrated that each bivariable comparison among these comfort variables were statistically significant (p’s < 0.01), with the exception of the difference between filling and disposing of prescriptions (p = 0.082).

**Table 1:** Descriptive statistics on parent–child conversations and prescription medication comfort.

	M	SD	*f*	*%*
Did your parents ever talk to you about…				
prescription medication safety?			99	59%
taking your prescription meds are prescribed?			141	84%
what to do with excess/leftover prescription meds?			38	23%
On a scale from 1 (not at all) to 10 (100% comfortable), how comfortable are you with…				
following prescription guidelines? filling a prescription on your own?	9.37 7.98	1.22 2.82		
disposing of meds when no longer needed?	8.32	2.45		

Note: n = 167.

### 3.2 Predicting youth comfort

A series of three multiple regression analyses were then performed predicting each of the three prescription medication comfort variables (see Table 2). Predictors included participant demographic characteristics (age, gender, race, ethnicity, and college GPA), participant recent prescription medication history, general parenting characteristics (mother–child and father-child relationship quality; parental active tracking), and specific parent–child discussions regarding prescription medications.

**Table 2:** Standardized regression coefficients and (p-values) predicting comfort with prescription medications.

	How comfortable are you with…		
	Following prescription guidelines?	Filling a prescription on you own?	Disposing of medications when no longer needed?
Age	−0.01 (0.937)	0.20 (0.024)	0.21 (0.011)
Gender (1 = Man: 2 = Woman)	0.25 (0.006)	0.18 (0.040)	0.19 (0.024)
Race (1 = White; 2 = Non-White)	0.11 (0.216)	0.12 (0.181)	0.16 (0.058)
Hispanic Ethnicity (1 = No; 2 = Yes)	0.10 (0.231)	−0.04 (0.614)	0.00 (0.964)
College GPA	−0.13 (0.138)	−0.19 (0.028)	−0.18 (0.032)
Recent Prescription Medication History	−0.09 (0.340)	0.04 (0.622)	−0.10 (0.236)
Mother-Child Relationship Quality	−0.06 (0.531)	−0.03 (0.766)	−0.06 (0.551)
Father-Child Relationship Quality	0.18 (0.047)	0.21 (0.019)	0.25 (0.004)
Parental Active Tracking	−0.01 (0.958)	0.06 (0.547)	0.06 (0.478)
Did your parents ever talk to you about… (1 = Yes; 2 = No)			
prescription medication safety?	0.00 (0.965)	0.01 (0.942)	0.10 (0.263)
taking your prescription medication as prescribed?	0.01 (0.955)	−0.00 (0.962)	0.04 (0.682)
what to do with excess/leftover prescription medication?	-0.130 (0.159)	−0.05 (0.569)	−0.24 (0.006)

Note: n = 142.

Results indicated that women tended to be more comfortable than men across all three outcomes, and older individuals tended to be more comfortable with filling and disposing of prescriptions than younger individuals. Greater GPA was associated with less comfort across all of the four outcomes (though only approaching significance for comfort following guidelines). Recent medication history was not associated with outcomes. General parent–child relationship quality was inconsistently associated with comfort outcomes, and parental active tracking was not associated with any outcomes. Regarding specific parent–child conversations regarding prescription medications, a discussion regarding medication disposal was the strongest predictor of comfort disposing of medications. This coefficient (*β* = -0.23) was actually the largest across all predictors and outcomes.

All Variance Inflation Factors across models were <1.50, indicating minimal potential for multicollinearity concerns. In order to further isolate the potential utility of parent–child discussion regarding disposal, we performed a supplemental analyses predicting comfort disposing of medications using all previous predictors and adding the two other comfort variables (following guidelines and filling prescriptions). The associations between a discussion of disposal and disposal comfort remained significant (*β* = −0.19, p = 0.011).

### 3.3 Follow-up analyses – Predicting disposal and retention practices

Since parental discussion of medication disposal was associated with comfort disposing of medications, we performed follow-up logistic regression analyses examining the extent to which this comfort is associated with appropriate disposal (i.e., use of a dedicated drop-off location) and inappropriate retention practices (see Table 3). Results indicated that use of a dedicated medication drop-off was predicted by prescription experience (both currently taking a prescription and past year frequency of prescriptions), greater parental active tracking efforts, and greater comfort disposing of excess medications. Retention of excess medications was predicted by White race, prescription experience, and lower comfort with medication disposal.

**Table 3:** Odds ratios and 95% confidence intervals predicting use of a medication drop-off location and retention of excess prescription medications.

	Uses a dedicated prescription medication drop-off location		Retention of excess prescription medications	
	Odds Ratio	95% CI	Odds Ratio	95% CI
Age	0.96	0.67–1.38	0.85	0.54–1.32
Gender (1 = Man: 2 = Woman)	0.89	0.33–2.46	0.75	0.29–1.93
Race (1 = White; 0 = Non-White)	1.60	0.43–5.96	**5.19**	1.11–24.34
College GPA	1.35	0.79–2.32	1.16	0.72–1.88
Currently taking a prescription	**4.52**	1.44–14.22	**0.33**	0.13–0.87
Frequency of prescriptions in past year	**1.76**	1.13–2.72	**0.62**	0.39–0.99
Parental Active Tracking	**2.17**	1.09–4.33	1.18	0.61–2.31
Comfort disposing of medications when no longer needed	**1.67**	1.22–2.30	**0.71**	0.59–0.84

Note: Bolded values indicate *p* < 0.05.

## 4 Discussion

The current study sought to investigate the role of parenting behaviors and communications on college student prescription medication knowledge and behaviors. Misuse of prescription medication is prevalent among college-aged youth with a variety of associated negative consequences. Opioid and antibiotic misuse were examined, with results indicating that (a) students frequently do not finish prescriptions as intended and (b) more than 1 in 7 students prescribed an opioid or antibiotic reporting retention of their excess medication. These practices increase risk for subsequent improper use (observed in 1 in 9 youth with an antibiotic prescription and 1 in 15 youth with an opioid prescription), as well as downstream negative effects like addiction, overdose, or antibiotic resistance.

The observed rates of inappropriate prescription medication behavior stand somewhat in contrast to high student self-reported comfort with prescription-related behaviors. The mean levels of comfort understanding and following prescription instructions and guidelines were each between 9 and 10 (with 10 meaning 100% comfortable). Reports were lower for comfort filling a prescription and disposing of excess medication (though means were still 8–8.3 out of 10). It is possible that less knowledgeable participants answered with intentional overconfidence, as patient understanding of medication instructions has been demonstrated to be quite poor in the general population (Hanchak et al., 1996, Wolf et al., 2011, Wolf et al., 2006). This explanation is somewhat supported by the fairly consistent finding that students with higher college GPAs reported lower levels of comfort.

Our multivariate models implied woman tend to be more comfortable with prescription-related activities and that parenting characteristics may play a role in determining youth prescription medication behaviors. Parent-child relationship quality inconsistently predicted comfort, while efforts by parents to track youth activities were not associated with comfort. These differential findings are, essentially, not surprising given that relationship quality has previously been linked with youth self-confidence (Bulanda and Majumdar, 2009), while tracking/monitoring lacks a clear mechanism for influencing comfort/confidence. Importantly, our study provided some evidence that domain-specific parent–child conversations may impact youth reported comfort with prescription medication-related behaviors.

In our study, the first two comfort variables examined (e.g., understanding prescription instructions, following prescriptions) implicitly tap youth perceptions of competence or ability to follow directions, such that domain specific discussions likely have limited relevance. The last two comfort variables, however, represent specific youth behaviors in which dedicated parental guidance may be beneficial. While we did not collect data on whether parents talked with their children about how to fill a prescription, we did find that specific discussions about appropriate disposal of excess medication was significantly predictive of comfort in this domain when accounting for general parenting. This finding echoes work showing that specific parental communications regarding alcohol use can be uniquely predictive of youth alcohol misuse (Chaplin et al., 2014, Lam et al., 2017, Napper, 2019).

These findings led us to perform subsequent analyses to see the extent to which youth perceptions of comfort disposing of medications (partially the result of parental specific discussion) is associated with actual behaviors. Our findings imply that comfort is independently associated with appropriate disposal of excess medications. These associations are directly in line with expectations based on Self-Determination Theory (Ng et al., 2012), with comfort serving as an analog for feelings of competence, which predict autonomous motivations and subsequent engagement in the behavior (e.g., proper disposal). While there are a variety of risk and protective factors for youth prescription misuse, identification of a clear, relatively simple, and, based on our data, likely modifiable behavior like proper disposal that can decrease risk is of considerable value. Improper disposal of excess medications is certainly not the primary risk factor for later misuse, but *the presented* findings highlight the potential utility of including parental discussions regarding medication safety and disposal in prevention programs seeking to mitigate youth prescription concerns.

### 4.1 Limitations

There were several limitations to the current study. First, the current study focused on prescription drug use, but a portion of adolescent and young adult populations have never been prescribed prescription medications. Second, data from this study was collected from a single university, and as a result, all participants were college students. With this study focusing exclusively on college youth, a subsequent sample including non-college youth might provide valuable insight. Therefore, more research with a larger and more diverse sample geographically and demographically is needed. Third, the current study focused exclusively on opioids and antibiotics, such that subsequent studies might benefit from examination of prescription stimulants, as well, given their prevalence among college-age youth. Fourth, a relatively large number of descriptive and inferential analyses were performed in the current study, such that independent replication would greatly support the validity of the current findings. Finally, the measurement of parent–child discussions regarding prescription medication was relatively crude and only from the student perspective. The items regarding prescription medication behaviors, though face valid, were developed for the current study. Future work would benefit from using a more nuanced measure of discussions from multiple informants and from incorporation of more thoroughly validated measures of prescription-related activities.

## 5 Conclusions

Prescription medication misuse is a significant public health concern impacting adolescents and young adults. The current study provides evidence for the roles parents might play in helping to prevent youth prescription-related harm, with particular focus on opioid and antibiotic misuse. Parent-child discussions targeting specific behaviors, like medication disposal, have the potential to mitigate youth prescription medication misuse.

### CRediT authorship contribution statement

**Caitlin C. Abar:** Conceptualization, Methodology, Formal analysis, Investigation, Resources, Writing – original draft, Writing – review & editing, Supervision. **Alexis Combs:** Investigation, Data curation, Formal analysis, Writing – original draft. **Allison Miley:** Investigation, Formal analysis, Writing – original draft. **Rebecca Ruffino:** Investigation, Formal analysis, Writing – original draft. **Beau Abar:** Conceptualization, Methodology, Writing – review & editing, Supervision.

## Declaration of Competing Interest

The authors declare that they have no known competing financial interests or personal relationships that could have appeared to influence the work reported in this paper.
